# The impact of integrating electronic referral within a musculoskeletal model of care on wait time to receive orthopedic care in Ontario

**DOI:** 10.1371/journal.pone.0241624

**Published:** 2020-11-03

**Authors:** Heba Tallah Mohammed, Lori-Anne Payson, Mohamed Alarakhia

**Affiliations:** 1 eHealth Centre of Excellence, Kitchener, Ontario, Canada; 2 Department of Family Medicine, McMaster University, Hamilton, Ontario, Canada; Western University, CANADA

## Abstract

An MSK model of care for hip and knee patients integrated with an electronic referral solution (eReferral) has been deployed within four subregions across Ontario. Referrals are sent from primary care offices to a central intake (CI), where the referral forms are reviewed and forwarded, if appropriate, to a rapid access clinic (RAC) where patients are assessed by an advanced practice clinician (APC). The pragmatic design of eReferral allows for a seamless flow of electronic orthopedic referrals from primary care to CI. It also enables CI to process and transcribe faxed referrals into the eReferral system for a smooth flow of data electronically to the RACs. In general, wait time is the time interval between receiving the patient’s referral at CI or the surgeon’s office until receiving the orthopedic surgeon's first consultation. Wait time is further broken down into wait 1 a and wait 1 b. Wait 1 a is the time between the receipt of the referral at CI until the date of the first initial assessment at the RAC. This study aimed at: a) assessing the processing time of orthopedic referrals at central intakes (CI) to be forwarded to the RAC, b) assessing the wait time (wait 1 a) of orthopedic referrals processed through the eReferral system to receive an initial assessment at the RACs. c) comparing the ability of the RACs to meet the target wait time for assessment (four weeks) by the method of referral (eReferrals vs. fax). d) evaluating patients’ satisfaction with the length of time they waited to receive care at the RACs with eReferral. We used Ocean eReferral database to access MSK hip and knee referral data processed through the system. Patients whose referrals were initiated electronically through the system and opted to receive email notification of their referral status had the opportunity to take an online satisfaction survey embedded in the booked appointment notification message. There were 1,723 patients initially referred electronically for hip, and knee pain consults, while 13,780 referrals started as paper-based and transcribed into the system to be forwarded later electronically by CI to a RAC. Higher mean processing time at CI by 21.76 days for paper-based referral was detected as opposed to referrals received electronically (p<0.001). RACs took significantly less time to book appointments for referrals initiated electronically with a shorter average wait 1a of 21.42 days for eReferrals compared to paper-based referrals (p<0.001). RACs timeframe to book an appointment was significantly shorter for eReferrals versus fax referrals. A total of 393 patients completed the patient satisfaction survey with a response rate of 16%. Overall, 87.7% were satisfied with their experience with the eReferral process, and 81% agreed that they had waited a reasonable time to receive the needed care. eReferral can elicit faster processing of referrals and shorter wait time for patients, which improved patient satisfaction with the referral process.

## Introduction

Musculoskeletal (MSK) disorders include degenerative joint disease, which affects millions in western nations [1]. Pain and limitations to physical activity are associated with MSK disorders [1]. Evidence shows that MSK disorders can cause significant disability and loss of productivity and often negatively impact the quality of life for MSK patients [1–3]. MSK patients require a suitable orthopedic assessment and management in order to function appropriately [1]; however, many Canadians usually experience a delay in accessing care. In some cases, patients had waited up to two years to receive an orthopedic consult appointment [4]. Research has shown that approximately 45% of MSK patients are not considered surgical candidates and would benefit more from receiving conservative management than waiting for the surgical consultation [5–8]. Lengthy wait periods and delays to access care impose a negative impact on patients’ health and quality of life [9].

In an effort to improve and expedite care for MSK patients, a new model of care for hip and knee has been implemented in care settings across Canada to support interdisciplinary collaboration [4, 10]. In Ontario, this model was further developed to include a dedicated central intake (CI) model for MSK referrals in 2017.

In this new MSK model of care, referrals are sent from primary care offices to a central intake (CI), where the referral forms are reviewed for missing information and triaged to the appropriate clinic according to the patients’ needs and orthopedic specialization. Unless the referral is urgent, it is forwarded to a rapid access clinic (RAC) where patients are assessed by an advanced practice clinician (APC), either a physiotherapist, or chiropractor [4, 10].The APC provides the first line of screening and assessment of orthopedic patients within four weeks of the original referral date [4, 10]. The extended scope of practice of an APC enables them to offer the patient conservative management strategies, and order appropriate diagnostic imaging. The APC also triages and prioritizes potential surgical candidates for an orthopedic surgical consult [4, 10]. Razmjou et al. (2013) reported a significant agreement on the APC’s assessment of patients with shoulder arthritis and the resulting diagnosis provided by the orthopedic surgeon [11]. APC’s screening and evaluation of MSK patients and the ability to provide effective management strategies in some cases have the potential to increase specialists’ capacity and potentially improve orthopedic wait times by decreasing the number of patients that need to be referred on for an orthopedic consult. Napier and colleagues reported that more than 75% of patients on the waitlist of their sampled patients were at the top position of a waitlist to see an orthopedic surgeon in British Columbia. At the same time, after the initial assessment of these patients, it was found that patients’ conditions would be effectively managed by a physiotherapist, which reduces the need to be placed on a surgical waitlist [12]. Similarly, Razmjou and colleagues’ findings showed a significant improvement in orthopedic shoulder patients’ conditions as 53% of the patients were managed adequately by an APC and did not require surgical consultation [11].

According to the Surgical Information Program Data Standardization Ontario Guide, the provincial wait time to receive orthopedic care for hip and knee patients for the MSK model consists of two distinct wait periods; wait time 1 and wait time 2 [13]. Overall, wait time 1 is the time interval between receiving the patient’s referral at CI or the surgeon’s office until receiving the first consultation from the orthopedic surgeon. Wait time 1 is further broken down into wait 1 a and wait 1 b. Wait 1 a is the time between the receipt of the referral at CI until the date of the first initial assessment by the APC at the RAC. While wait 1b, is the time between the date the APC refers the patient for an orthopedic consultation (usually decided at the initial assessment date) until the first surgeon consultation. Wait 2 is the time between the orthopedic surgeon referring the patient for surgery and the actual date of surgery [13]. Referral time to an orthopedic consult varies by the practicing clinics. A study conducted in Alberta reported that missing information prolongs the referral processing time, therefore adding as many as 46 days to the wait time for patients to receive orthopedic surgical consultation [9]. Timely communication of complete adequate information is an elemental factor for an efficient process, and it requires a remarkable effort to achieve [14, 15]. One approach to supporting communication and interoperability of information within the healthcare sector and potentially addressing some of the challenges noted above is by leveraging external resources such as an integrated digital model [14, 15].

**Fig 1:**
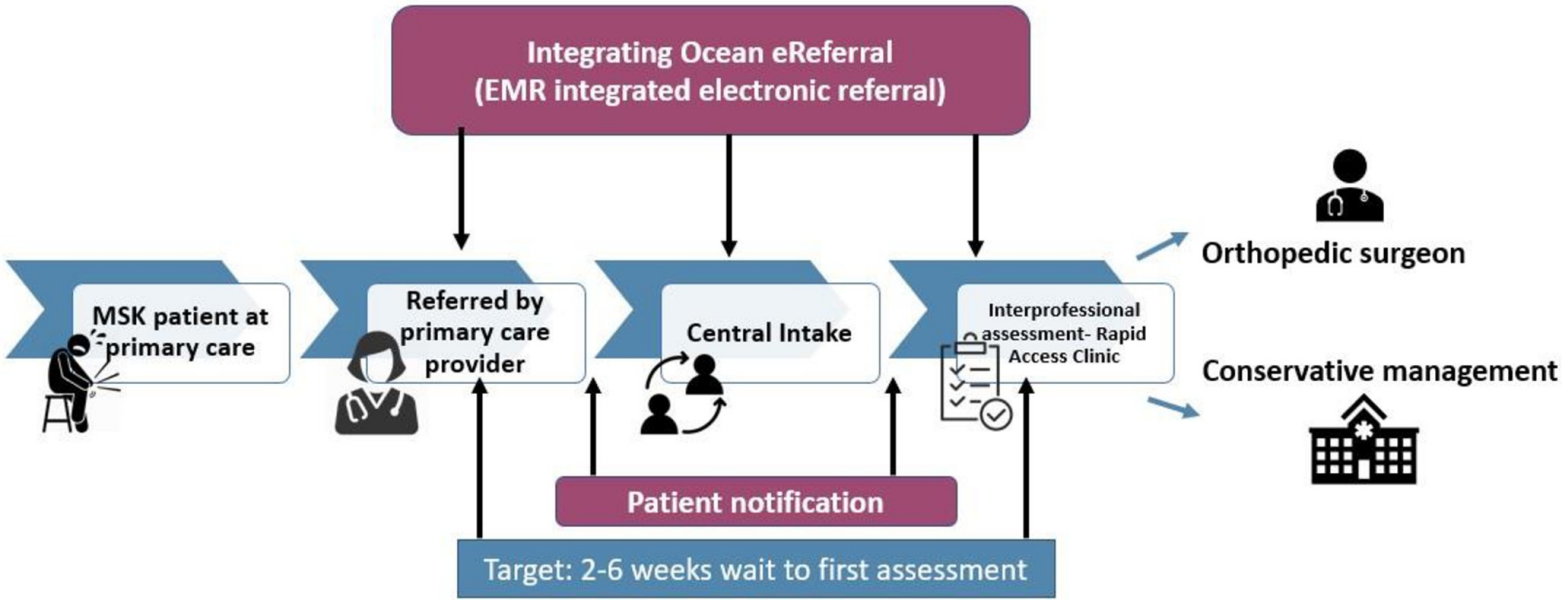
Integrating eReferral Ocean system and MSK model of care.

At the same time as the new MSK model of care was rolled out across Ontario, the eServices Program–formerly known as the System Coordinated Access (SCA)–was funded by the Ministry of Health (MOH) to deploy the Ocean eReferral network (eReferral) solution. eReferral is an electronic solution that has been actively used across many subregions across Ontario–Waterloo Wellington (WW), Erie St. Clair (ESC), South East (SE), North East (NE), Champlain, South West (SW), and Hamilton Niagara Haldimand Brant HNHB–to support patient access to care for various health pathways. The eReferral solution has been deployed to enable the hip and knee MSK model of care since 2018 in four subregions (WW, ESC, SE, and NE) (Fig 1). The pragmatic design of eReferral allows for a seamless flow of electronic orthopedic referrals from primary care to CI. It also enables CI to process and transcribe faxed referrals into the eReferral system for a smooth flow of data to the RACs and orthopedic surgeon offices. eReferral features enable CI to detect and record any missing referral information. Also, eReferral allows for secure and timely communication with the providers using a standardized in-application messaging feature. When the electronic referral is forwarded to the RACs, the APCs can accept and triage it and book the appointment using the eReferral solution. Having the appointment entered into the solution allows for the automatic calculation of wait 1a. The APCs can also document outcome notes from their assessment appointments and communicate effectively and efficiently with providers using eReferral secure messaging.

According to the Ontario Ministry of Health (MOH), Digital Health Playbook–a document designed to help Ontario Health Teams build an efficient digital health plan within the health sector–the digital health infrastructures equip clinicians with the necessary tools and assets to streamline workflow and access to care [16]. Electronic referring of patients facilitates a streamlined process and helps to reduce processing time compared to the traditional referral methods [17]. For example, Ocean eReferral ensures legible and completed referral forms to be sent through the system, saving time spent by administrative staff tracking down missing information. Ocean eReferral secure in-application messaging, status tracking and online appointment confirmation features make communication, gathering of information, scheduling of appointments, and the tracking of referrals easier and quicker between providers and patients. Ocean eReferral grants access to a health map directory for the general public, an enabler of evidence-informed decision making. On this health map directory, the eReferral solution allows for the displaying of real-time wait times information to access a care by service and specialist. Ocean eReferral also grants clinicians who adopted the system access to an encrypted and secure Ocean eReferral database free of charge. This database allows clinicians to download and review eReferral patient data processed through their clinics. Furthermore, Ocean eReferral provides additional features for patients. It allows patients to track their referral process and receive email notifications throughout their referral. As part of the eServices Program’s ongoing evaluation of the eReferral system, the notification email of patients’ booked appointment has an embedded link to a satisfaction survey that collects patients’ perspective and satisfaction level of the referral process and wait time to access care.

To date, many studies have provided perspectives on the lengthy wait times to access orthopedic care in Canada [18, 19], but to our knowledge none has addressed the amount of time spent by CI staff to process orthopedic referrals, or assessed patient wait times to access orthopedic care in the MSK hip and knee model of care using an electronic referral solution.

**Fig 2:**
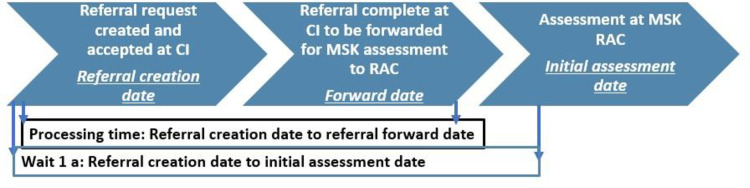
Definition of referral requests dates and wait time to access assessment centres in the MSK model.

The primary objective of this article is to:

- assess the wait time (wait 1 a) for a hip or knee assessment with an APC participating in the integrated eReferral- MSK model (Fig 2).
- compare the ability of the RACs to meet the target wait time for assessment (4 weeks) by the method of referral—initially referred electronically versus the traditional faxed based methods across four subregions in Ontario (WW, ESC, SE, and NE).

This study also aimed to:

- evaluate the time spent processing referrals at CI from the time they receive a referral from primary care until it is forwarded for an APC consult (Fig 2).
- explore hip and knee patients' perspectives of the eReferral process and time they have to wait to access care at a RAC.

Orthopedic specialists within the subregions have not fully adopted the eReferral solution yet. Therefore, it is out of this article's scope to assess wait 1 b and wait 2 to access specialist care and make comparisons by referral methods.

## Material and methods

### Study design and data sources

This is a descriptive study that assesses the processing time of orthopedic referrals at CI, the wait time of orthopedic hip and knee referrals to access care at an assessment centre by the method of referral and evaluates patients’ perspective on wait time using Ocean.

#### Ocean eReferral network database

This study used Ocean eReferral Network database to access and collect anonymous referral data of hip and knee MSK referrals that were processed through the system from primary care offices to the RACs for an initial assessment. In Ocean, orthopedic hip and knee referral data is either populated directly from the primary care physicians’ electronic medical records (EMR) into Ocean solution and stored in Ocean electronic database, or manually transcribed from paper-based referrals into the system by a CI staff. Subsequently, the transcribed data is also stored in the Ocean electronic database. This database is encrypted, secure and routinely accessed, evaluated and maintained by the eServices Program Benefits Realization (BR) Evaluation team and the Ocean solution vendor team. This allows for accessing accurate creation dates of the referral request, forward date of the referral to the first assessment at the RAC, and the initial appointment date at the RAC. The Ocean solution is designed to extract anonymous data from the referral source. Patient referral data is linked to a unique, encoded number automatically created by Ocean. No direct patient identifiers can be extracted or accessed from the Ocean analytics database. Hip and knee referral data were collected from January 1, 2018, when the integration of the MSK model and eReferral began, to the end of January 2020. The referral data were collected from the four subregions across Ontario (WW, ESC, SE, and NE) that have adopted the integrated model for hip and knee MSK referrals.

#### Patient satisfaction survey

Patients whose referrals were processed electronically through the Ocean system and provided consent to their clinicians to receive notification of their referral status had the opportunity to join our online satisfaction survey. With the booked appointment notification, an invite, and a link to the survey are embedded in the email message. The survey questions were developed based on a systematic approach consisted of

1. an extensive literature search of patients’ satisfaction of services in the healthcare sector,
2. testing of survey questions, excluding redundant items and comprehensive reviews from authors,
3. seeking feedback from the patient and caregiver working group in the Waterloo-Wellington subregion, and
4. further modification and formatting based on the group feedback (More details about assessing patients survey questions are available through a published article) [20].

Survey data is hosted and stored in the Ocean research database, which is monitored and evaluated by the eServices BR team. This ongoing online survey is conducted as part of the formative evaluation of the eReferral system. All patient data is anonymous, and patients provide answers only to questions they choose to respond to them. The patients' responses were also collected from January 1, 2018, until the end of January 2020.

### Data abstraction and sample

#### Wait time data

A total of 15,503 hip and knee MSK patients with orthopedic referral data and scheduled appointment for an assessment at the RAC were collected in this study. The BR evaluation team extracted the data from Ocean eReferral database (first and second authors of this article) during the first week of February 2020. The eServices Program evaluation team has administrative access to the secure Ocean sub-regional eReferral database across Ontario and has clearance to download the eReferral data from each site.

In general, for patients’ eReferral data to be eligible for inclusion in the study and analysis, referrals must fulfil the following requirements:

1. referrals must be for hip and knee joints and are processed through Ocean either electronically or transcribed from a fax,
2. referrals must be processed through CI to be forwarded to a RAC for an initial assessment, and
3. referrals must have a scheduled booked appointment for an assessment at the RAC.

Referrals for reasons other than hip and knee complaints, not processed through Ocean, and through CI to be referred for an initial assessment at the RAC or had not been scheduled for an assessment at the RAC were excluded from the data set and analysis.

The following indicators were extracted from the eligible referrals for analysis: a) patient referral, forward and appointment/assessment date stamps, b) patient demographic characteristics like age, gender, city, and local health service planning area c) method of referral, d) destination of the referral, e) referral status, and e) wait 1 a. Ocean database automatically calculates wait time in days using the designated referral dates. Wait 1 a was calculated based on the time interval between the patient’s referral creation date and scheduled initial assessment date with an APC at the RAC. When patient-related reasons that affect patients’ readiness to receive a consult are added to the referral note, the system automatically deducts the time when patients are unavailable from the wait time calculation.

In addition, we also collected the APC initial assessment outcome note of whether the patient was deemed a surgical candidate or not–an indictor extracted into the database and referral data–in correspondence to the method of referral from the regions that collect such data (ESC, SE, and NE).

Also, processing time was calculated by the analysis team based on the time interval between the referral creation date and the forward date of the referral from CI to the RAC.

Average wait 1a, processing time, and the ability to meet the target wait time for assessment (four weeks) at RACs were compared for hip and knee referrals in correspondence to the method of referral.

#### Patient satisfaction data

For the responses on patient satisfaction, all orthopedic hip and knee patients who responded to the invite to complete the satisfaction survey were included. Those who were referred for reasons other than MSK hip and knee were not part of the analysis. A total of 393 hip and knee MSK patients’ responses were collected. The survey consists of 15 questions that assess satisfaction with eReferral. Participants were invited to rate their satisfaction and agreement levels using a 5-point Likert scale. They were also asked to provide their feedback in an open-ended comment box following each rating question. The survey also collected patients’ demographics such as age, gender, city, and referral request type. Rating questions included: patients’ satisfaction level with the electronic referral process, and their wait time; agreement level that different options were provided for a referral to either a specific specialist or hospital; whether patients’ choices were considered; and if they felt more informed throughout the electronic process (More details about patients survey questions are available through a published article) [20]. Besides the type of referral request and the responses to the patients’ demographic questions, only the following questions were directly related to the topic and included in the study: 1) Please rate your overall satisfaction with this electronic referral process. Responses included strongly satisfied, satisfied, neither satisfied nor dissatisfied, dissatisfied, and strongly dissatisfied. 2) In general, I feel that I was able to get the care I needed in a reasonable amount of time. Responses included strongly agree, agree, neither agree nor disagree, disagree, and strongly disagree. These questions were chosen as indicators for patients’ satisfaction with the eReferral system and wait time to access orthopedic care.

This descriptive quality improvement study was approved for an exemption of ethics review by the Ethical Review Board of McMaster University (HiREB).

### Statistical analysis

Data from all hip and knee referrals processed through Ocean and patients’ responses to the survey questions were managed and analyzed using the Statistical Package for Social Sciences (SPSS) (SPSS; IBM Corp, Armonk, New York. Version 26; 2020). Descriptive analyses were conducted, and summary statistics were illustrated as frequencies (%) or mean (SD). The average wait and processing times in days were calculated as well as the 90th percentile. Independent student t-test for continuous variables (wait and processing times) and Chi-square analyses for categorical variables were used to examine the difference in patients’ characteristics (age, gender, subregion, and joint affected) and to assess the time (in weeks) to book an appointment at the RAC. The assumption of homogeneity of variance by the method of referral was violated in some variables as assessed by Levene’s test for equality of variance. Therefore, Welch’s t-test was computed to determine if there were differences in the means of processing time and wait 1 a between Ocean eReferrals and paper-based referrals. Also, post hoc analysis involved pairwise comparisons using multiple z-tests of two proportions with a Bonferroni correction was used to test the ability to meet the target wait time for assessment and the length of time in weeks to book an appointment at the RAC. A 2-sided P value of *<*0.05 was used as the cut-off for statistically significant differences. Original P values are shown in the results tables, and footnotes indicate whether the P levels were <0.01 after the adjustment.

## Results

Data was collected from 15,503 MSK hip and knee patients’ referrals that met the inclusion criteria, as mentioned above, across all four subregions over two years.

### Demographic characteristics

The demographics and characteristics of these patients are summarized in Table 1. Overall, the mean age of patients was 66.28 years. More than half of patients (56.3%) were females and 43% were from the north subregion. More than two-thirds of patients (68.6%) were referred for a knee pain consult Table 1.

**Table 1:** Characteristics of patients with processed referral in Ocean by method of referral.

Characteristics N (%)	All Patients N = 15503	Ocean eReferral N (%)	Paper-based Referrals N (%)	P value (eReferral vs. Paper-based)
		1723 (11)	13780 (89)	
**Gender, N (%)**				P = 0.306
Male	6732 (43.7)	694 (40.3)	6038 (43.8)	
Female	8771 (56.3)	1029 (59.7)	7742 (56.2)	
**Age**				P = 0.602
Mean (SD)	66.28 (15.5)	67.73 (14.4)	66.09 (16.6)	
**Subregion N (%)**				P< 0.001*
ESC	2671 (17.2)	59 (3.4) ^a^	2612 (18.9) ^b^	
NE	6606 (42.6)	353 (20.5) ^a^	6253 (45.7) ^b^	
SE	3396 (21.9)	110 (6.4) ^a^	3286 (23.7) ^b^	
WW	2830 (18.3)	1201 (69.7) ^a^	1629 (11.7) ^b^	
**Joint affected N (%)**				P = 0.109
Hip	4889 (31.5)	519 (30.1)	4370 (31.7)	
Knee	10614 (68.6)	1204 (69.9)	9410 (68.3)	

*Significant difference at p< 0.05; Non-significant difference at p≥ 0.05.

*Significant difference using Post hoc analysis using Bonferroni correction was accepted at *p* <0 .01. Different letters between groups = Significant difference (p<0.01); same letters between group means = Non-significant difference (p≥0.01).

Differences in the proportions of paper-based referrals vs. Ocean eReferral based on gender, age, and joints affected were not significant. However, significant differences were detected between both groups regarding distribution per subregion (p<0.001) Table 1.

### Processing and wait times

There were 1,723 patients initially referred electronically for hip and knee pain consults, while 13,780 referrals started as paper-based and transcribed into the system to be forwarded later electronically by CI for an APC consult. The mean processing times at CI and wait 1 a, in days, from primary care to APC consult, are presented in Table 2. Higher mean processing time at CI by 21.76 days for paper-based referral was detected as opposed to referrals received electronically. RACs took significantly less time to book appointments for referrals initiated electronically with a shorter average wait 1a of 50.30 days for eReferrals compared to 71.72 days for paper-based referrals. RACs timeframe for appointment was significantly shorter for eReferrals versus fax referrals. About three-quarters of eReferral (74%) were booked within the four-week timeframe of receiving the referral at the RAC, while only 56% of paper-based referrals were booked within the four-week timeframe.

**Table 2:** Comparison of wait times by method of referral and body part.

Wait time	Ocean eReferral N = 1723	Ocean Hip eReferral N = 519	Ocean Knee eReferral N = 1204	Ocean Paper-based Referrals N = 13780	Ocean Hip Paper Referrals N = 4370	Ocean Knee Paper Referrals N = 9410	P value (Overall eReferral vs. Paper referrals)
**Processing time at CI**							**P<0.001**
*Mean (days)*	3.5	3.3	3.5	25.2	23.1	26.2	
*90*^*th*^ *percentile*	7.0	6.0	7.0	58.0	24.9	77.0	
**Wait 1 a**							**P<0.001**
*Mean (days)*	50.3	46.9	51.7	71.7	66.3	74.2	
*90*^*th*^ *percentile*	109.0	100.0	111.0	349.9	199.9	364.0	
**Booked an appointment at RAC N (%)**							P<0.001*
Within 4 weeks	1278^a^ (74.2)	397 (76.4)	881 (73.1)	7743^b^ (56.1)	2474 (57.0)	5269 (56.0)	
Within 6 weeks	1463 ^a^ (85.0)	440 (85.0)	990 (82.2)	9763 ^b^ (71.0)	3081 (70.4)	6682 (71.0)	
Within 12 weeks	1622 ^a^ (94.1)	495 (95.3)	1127 (94.0)	11798 ^b^ (86.0)	3706 (85.0)	8092 (86.0)	
Within 24 weeks	1712 ^a^ (99.3)	517 (99.6)	1195 (99.2)	12256 ^b^ (89.0)	3882 (89.0)	8374 (89.0)	
Within 36 weeks	1723 ^a^ (100)	519 (100)	1204 (100)	12358 ^a^(90.0)	3889 (89.0)	8469 (90.0)	
Within 48 weeks				13732 (99.6)	4337 (99.2)	9395 (99.8)	
Within 96 weeks				13780 (100)	4370 (100)	9410(100)	
**Surgical candidate Yes**	864 (50.1)	269 (51.8)	595 (49.4)	7284 (52.8)	2425 (55.5)	4859 (51.6)	P = 0.151

*Significant difference at P< 0.05; Non-significant difference at p≥ 0.05.

* Significant difference using Post hoc analysis using Bonferroni correction was accepted at *p* <0 .01. Different letters between groups = Significant difference (p<0.01); same letters between group means = Nonsignificant difference (p≥0.01).

No significant difference was detected on the APC consultation outcome concerning the method of referral. Only half of the eReferral cases and paper-based referrals seen by APC were deemed to be surgical candidates (50.1% and 52.8%, respectively) Table 2.

### Patient satisfaction

Of the 15,503 referred patients, 2,469 patients provided their consent and received an automated e-mail notification of their booked appointment, which included an invite to complete the survey. A total of 393 patients completed the patient satisfaction survey with a response rate of 16%. More than three-quarters of patients (78.5%) were 60 years of age or older, and almost two-thirds (62.3%) were females. The majority of respondents (91.9%) were from the ESC and WW. The demographic characteristics of the knee and hip patients were not statistically different.

Overall, the majority of patients were satisfied/very satisfied (87.7%) with their experience with the eReferral process, and 81% agreed/strongly agreed that they had waited a reasonable time to receive the needed care with the MSK- eReferral model. No significant differences were detected with regard to gender, satisfaction with the eReferral process, and agreement that wait time to receive care was reasonable between hip and knee patients Table 3.

**Table 3:** Characteristics of the surveyed patient and level of Satisfaction of eReferral process and MSK wait time.

Characteristics	Overall hip and knee eReferrals	Hip eReferral	Knee eReferral	P value
	N (%)	N = 126	N = 267	Hip vs. knee
***Patients age***				P = 0.257
• ≤ 29	10 (2.5)	3 (2.4)	7 (2.6)	
• 30–39	3 (0.7)	1 (0.8)	2 (2.6)	
• 40–49	11 (2.8)	1 (0.8)	10 (3.7)	
• 50–59	61 (15.5)	15 (11.9)	46 (17.2)	
• 60–69	144 (36.7)	47 (37.5)	97 (36.3)	
• ≥ 70	164 (41.8)	58 (46.5)	106 (39.7)	
***Patient gender***				P = 0.635
• Male	146 (37.8)	51 (41.2)	95 (36.2)	
• Female	241 (62.3)	73 (58.8)	168 (63.8)	
***Subregion***				P = 0.415
• ESC	209 (53.2)	76 (53.2)	142 (53.2)	
• WW	152 (38.7)	52 (41.3)	100 (37.5)	
• NE	4 (1)	0 (0.0)	4 (1.5)	
• SE	28 (7.1)	7 (5.6)	21 (7.9)	
***Satisfied with eReferral process***				P = 0.455
• Very satisfied	195 (50.3)	60 (47.6)	135 (50.5)	
• Satisfied	144 (37.4)	50 (39.6)	94 (35.2)	
• Dissatisfied	14 (3.6)	2 (1.6)	12 (4.5)	
• Very Dissatisfied	34 (8.7)	11 (8.7)	23 (8.6)	
***Reasonable wait time to receive care***				P = 0.204
• Strongly agree	100 (28.0)	33 (26.1)	67 (25.1)	
• Agree	190 (53.0)	67 (53.1)	123 (46.1)	
• Disagree	55 (15.0)	12 (9.5)	43 (16.1)	
• Strongly disagree	15 (4.0)	3 (2.4)	12 (4.5)	

## Discussion

This research is the first published study providing detailed information on the orthopedic hip and knee referral wait times within the MSK model by method of referral. This study’s primary aim was to evaluate and compare the average wait time in days for hip and knee patients to access the MSK model of care by the method of referral. We also sought to assess patients’ satisfaction with the length of time they waited to access care. This study used standardized definitions of referral dates and processes to measure wait times for hip and knee patients accordingly. Overall, the quantitative data presented in this study has shown the positive influence of the referral technology on patient access to care.

With respect to the integration of the Ocean eReferral solution with the MSK model of care—a significant difference was noted between referrals initiated electronically and paper-based referrals for both the processing times of referrals at central intake as well wait time for patients to see an APC for an initial assessment (P<0.001). On average, it took central intake less time (21.76 days) to process the electronic referrals and forward it to the assessment center. Electronic referral forms are legible and more complete when sent from primary care offices to central intake, which may have contributed to this finding. Digital tools are recognized for their ability to efficiently use resources at the practice site and reduce administrative staff workload [21]. Integrating digital tools within clinical practice settings presents new opportunities to increase operational efficiencies and manage the delivery of care [18]. The less time CI staff utilizes to track down missing information, the earlier and faster they can forward the referral to the assessment centres. Our findings concur with Fyie and colleagues’ results from a study conducted in Alberta, 2013, which investigated the work processes and practices associated with the orthopedic hip and knee’s long wait times. They reported that involuntary system delays in the form of incomplete and inappropriate referrals to the orthopedic surgeons extensively consume time and significantly contribute to the prolonged wait time in accessing orthopedic care. About 20% of the referrals received at the surgeon’s offices are incomplete and missing required information, which results in an initial rejection of the referral, hence longer wait time for patients [9]. Furthermore, Hagglund and colleagues reported that integrating a standardized digital tool to share information leads to increased efficiency within and across healthcare organizations. It saves time that is often consumed in searching for information, through phone call and fax messages, and helps to attain the relevant information quickly at the point of need [22].

eReferral also gives patients the ability to receive appointment information by email and confirm the booked appointment online. Accordingly, the earlier the referral is received and accepted, the faster the appointment is scheduled at the assessment centers. This saves time for administrative staff to contact patients and support the efforts to reduce wait time. Our data shows that patients waited less time to access care at assessment centers by an average of 21.42 days with eReferral. In our study, the improvement in system efficiency is evident in the RACs ability to receive and book appointments for hip and knee patients faster and within shorter time frames with the eReferral solution. Patients initially referred electronically were booked within four weeks to a maximum of 9 months compared to up to 24 months with paper-based referrals.

Obtaining the patients’ perspective on the length of time to access care and their level of satisfaction with the referral system is essential to early assessment of a new integrated model of care. Overall, patients’ responses to the survey were positive about the electronic referral process. Hip and knee respondents demonstrated a high level of satisfaction with the electronic referral process and the length of time they had to wait to receive the MSK care with an overall satisfaction rate of 87.7% and 81%, respectively. No significant difference was detected between hip and knee patients related to their satisfaction with the eReferral process or length of wait time. This suggests a similarly high level of satisfaction among both conditions. A study conducted by Kreitz and colleagues in 2016 reported that patients’ overall satisfaction with the referral experience could be adversely affected by an additional 15 minutes of wait time from their scheduled appointment to meeting with a clinician [23]. Patient-provider communication technology, including appointment booking systems, is considered an elemental factor in providing timely access to health services [21]. Therefore, this notable decrease in wait time to receive a consult and the reported high level of satisfaction of our hip and knee patients’ with the length of wait time to access care can be an indicator of the success of the integrated model in supporting patients’ access to care within a reasonable time.

The MSK model–referring orthopedic patients to APC for initial assessment through CI–has proven its efficiency in triaging surgical candidates [5–8]. In our study, there was no significant difference in APC’s assessment outcome by referral method. Only about half of both hip and knee patients were identified as surgical candidates to be further referred to an orthopedic surgeon for a consult. This finding is consistent with previous research studies indicating that over half of patients waited unnecessarily for a surgical consult [9], and 40–60% of referred orthopedic patients can be managed effectively using conservative methods at the assessment center [11]. This process can substantially save surgeons’ time, shift their focus towards the clinical role, and shorten their waitlist [18].

The main focus of the healthcare system is on improving coordination within and efficiency of the healthcare sectors to enhance patient care [24]. The integration of a standardized digital structure such as Ocean eReferral that supports a recognized effective mechanism for interdisciplinary collaboration like the MSK care model can be the practical approach to achieving the ultimate goal. Our findings agree with studies that show that an integrated hybrid model within the health care system allows for enhanced transfer of and timely access to information that expands the health system capacity and offers more synchronized and optimized care coordination [22, 25].

### Limitations/strengths

This study's scope was somewhat limited to assessing the impact of the integrated model on the wait time to receive an initial assessment from an APC. Orthopedic specialists are still in the process of adopting Ocean eReferral solution. Therefore, it was difficult to obtain a large sample of wait time data of orthopedic surgeon clinics using Ocean. It is essential to continue the evaluation across various receiver groups and explore the impact of the integrated model on wait 1b and 2.

Ocean has recently added the ability to extract data from the central intake referral note to understand the reasons behind the missing information of orthopedic referrals. As this is a new feature and has not been widely utilized across all regions, it was impossible to allocate the causes of the missing information and the level of completeness of referrals to the method of referral. Once this indicator is well used within this hybrid model, it would be important to explore more to inform efforts to reduce the level of missing information.

As the patients’ survey was anonymous, we were unable to associate the patients’ perspective on wait time in this study and their actual wait time calculated in Ocean. Also, no comparison of patients’ responses was collected from patients whose referrals were initiated by fax.

The response rate to the patient survey was 16%. However, this proportion reflected only patients who opted to receive email notification of their referral process, which might have limited the received feedback and inflated the response rate. Potential selection bias is another limitation. Feedback might have been mainly obtained from participants who have access and more adaptive to using electronics, especially among the older age group most affected by osteoarthritis. Moreover, responses could be collected more from those influenced positively or negatively by the referral process. This may limit the generalizability of our findings. However, it is worth noting that our results are the first to highlight hip and knee patients’ insights about the electronical referral process and wait time of the integrated MSK model of care. Our findings could support efforts, currently in process by the eServices program’s change management team at primary care settings, promoting the eReferral email notification for more transparent access to care for patients.

Our study's strength lies in the relatively large sample size of 15,503 patients' wait time data from different orthopedic assessment practice settings using Ocean within four subregions across Ontario. Our results could be generalized across the province to some degree. This is due to the fact that our study accounted for the homogenous presence of a central intake system for orthopedic referrals, standardized rules for wait times, and screening practice at central intakes to forward referrals to RACs across the province while assessing the impact of the integrated design on processing and wait times.

### Directions for future research

Further investigation is essential to determine the impact of eReferral on other wait times data within the healthcare system to promote the broader implementation of the integrated models for enhanced access to care. Further explorations of patients’ perspectives of orthopedic wait times using the eReferral model with a larger sample size is necessary.

## Conclusions

Evidence from our study highlights the efficiency of integrating the Ocean eReferral solution with the MSK model of care. The process is shown to elicit faster processing of referrals and shorter wait time for patients. The process has further appeared to reinforce patients’ appreciation of the electronic referral model and the length of time they have to wait to access care.

## Supporting information

S1 Data Orthopedic wait time with eReferral and MSK. (XLSX)

S2 Data Patient satisfaction with eReferral and MSK model wait time. (XLSX)

## References

[1] Reginster Y. The prevalence and burden of arthritis. Rheumatology. 2002;41(1):3–6. 10.1093/rheumatology/41.S1.3 12173279

[2] Roux C, Guillemin F, Boini S, Longuetaud F, Arnault N, Hercberg S, et al Impact of musculoskeletal disorders on quality of life: an inception cohort study. Ann Rheum Dis. 2005; 64:606–611 10.1136/ard.2004.020784 15576417 PMC1755431

[3] Tüzün E. Quality of life in chronic musculoskeletal pain. Best Practice & Research Clinical Rheumatology. 2007; 21(3): 567–579 10.1016/j.berh.2007.03.001 17603000

[4] Desmeules F, Toliopoulos P, Roy J, Woodhouse L, Leroux M, et al Validation of an advanced practice physiotherapy model of care in an orthopaedic outpatient clinic. BMC Musculoskeletal Disorders. 2013; 14:162:1–10 23656928 10.1186/1471-2474-14-162 PMC3658921

[5] Hattam P. The effectiveness of orthopaedic triage by extended scope physiotherapists. Clinical Governance: An International Journal. 2004; 9(4):244–252.

[6] Heywood J. Specialist physiotherapists in orthopaedic triage-the results of a military spinal triage clinic. Journal-royal army medical corps. 2005; 151(3):152 10.1136/jramc-151-03-04 16440957

[7] MacKay C, Davis AM, Mahomed N, Badley E, et al Expanding roles in orthopaedic care: a comparison of physiotherapist and orthopaedic surgeon recommendations for triage. J Eval Clin Pract. 2009; 15(1):178–183. 10.1111/j.1365-2753.2008.00979.x 19239599

[8] Rabey M, Morgans S, Barrett C. Orthopaedic physiotherapy practitioners: Surgical and radiological referral rates. Clinical Governance: An International Journal. 2009; 14(1):15–19.

[9] Fyie K, Frank CY, Noseworthy T. Evaluating the primary- to-specialist referral system for elective hip and knee arthroplasty. Journal of evaluation in clinical practice. 2014; 20:66–73. 10.1111/jep.12080 24004242

[10] Aiken A, Harrison M, Hope J. Role of the advanced practice physiotherapist in decreasing surgical wait times. Healthcare Quarterly. 2009; 12(3): 80–83 10.12927/hcq.2013.20881 19553769

[11] Razmjou H, Robarts S, Kennedy D, McKnight C, Macleod A, et al Evaluation of an advanced-practice physical therapist in a speciality shoulder clinic: diagnostic agreement and effect on wait times. Physiotherapy Canada. 2013; 65(1): 46–55 10.3138/ptc.2011-56 24381382 PMC3563377

[12] Napier C, McCormack R, Brooks-Hill A. A physiotherapy triage service for orthopaedic surgery: An effective strategy for reducing wait times. Physiotherapy Canada. 2013; 65(4):358–363. 10.3138/ptc.2012-53 24396164 PMC3817883

[13] Cancer Care Ontario. Access to Care: MSK Rapid Access Clinic (RAC) Data Guidance document. 2018.

[14] Product Lifecycle Management for a Global Market: 11th IFIP WG 5.1 International Conference, PLM 2014, Yokohama, Japan, July 7–9, 2014, Revised Selected Papers.” Vol. 442. Berlin, Heidelberg: Springer Berlin Heidelberg, 2014.

[15] Paquette-Warren J, Vingilis E, Greenslade J, Newnam S, et al What do practitioners think? A qualitative study of a shared care mental health and nutrition primary care program. International Journal of Integrated Care.2006; 6 Available from: http://yywww.ijic.orgy. 10.5334/ijic.164 17041680 PMC1602056

[16] Ministry of Health. 2019. Ontario Health Team: Digital Health Playbook. Accessed on Jan 2020: http://health.gov.on.ca/en/pro/programs/connectedcare/oht/docs/dig_health_playbook_en.pdf.

[17] Cannaby S, Westcott D, Pedersen C, Voss H, Wanscher E et al. The cost benefit of electronic patient referrals in Denmark: full report. ACCA and MedCom in collaboration with the European Commission Information Society Directorate–General; 2005.

[18] Barua, B., & Fathers, F. Waiting your turn: Wait times for health care in Canada, Fraser Institute. report. 2014

[19] Liddy C, Nawar N, Moroz I, Mcrae S, Russell C, et al Understanding Patient Referral Wait Times for Specialty Care in Ontario: A Retrospective Chart Audit. HealthCare Policy. 2018;13 (3):59–69 10.12927/hcpol.2018.25397 29595437 PMC5863870

[20] Mohammed HT and Huebner LA. Assessing Patient Satisfaction and Experience With an Electronic Referral Process."Quality Management in Healthcare. 2020 29(1):20–29. 10.1097/QMH.0000000000000235 31855932

[21] Idowu A, Adeosun O and Williams K. Dependable online appointment booking system for NHIS Outpatient in Nigerian Teaching Hospitals. International Journal of Computer Science & Information Technology. 2014; 6(4): 59–73

[22] Hagglund M, Mostrom D and Koch S. Bridging the gap: a virtual health record for integrated home Care. International Journal of Integrated Care. 2007; 7(27): ISSN 1568-4156. 10.5334/ijic.191 17637872 PMC1919412

[23] Kreitz T, Winters B and Pedowitz D. The influence of wait time on patient satisfaction in the orthopedic clinic. Journal of patient experience. 2016; 3(2): 39–42. 10.1177/2374373516652253 28725834 PMC5513615

[24] Lorenzo V and Vázquez Navarrete M. Barriers and facilitators to health care coordination in two integrated health care organizations in Catalonia (Spain). Gaceta sanitaria. 2007; 21(2): 114–123. Web. 10.1157/13101037 17419927

[25] Whyte J. Managing digital coordination of design: emerging hybrid practices in an institutionalized project setting. Engineering project organization journal. 2011;1(3): 159–168.

